# Supplementary material for: Intratumoral CD40 activation and checkpoint blockade induces T cell-mediated eradication of melanoma in the brain
Source: Nat Commun. 2017 Nov 13;8:1447. doi: 10.1038/s41467-017-01572-7 (PMC5682289; doi:10.1038/s41467-017-01572-7)
Supplement: Supplementary file 1 — Supplementary Information [file 41467_2017_1572_MOESM1_ESM.pdf]

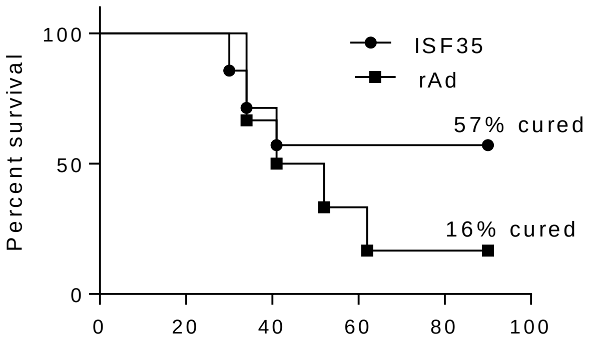

Supplementary Figure.1. Intratumoral ISF35 suppresses BP melanoma. Mice bearing 7-day s.c. BP tumors (derived from $BRAF^{V600E}xPTEN^{-/-}$ melanoma prone-mice) were treated as indicated, graph depicts long term survival(n=7). Survival analysis was performed with the log-rank test

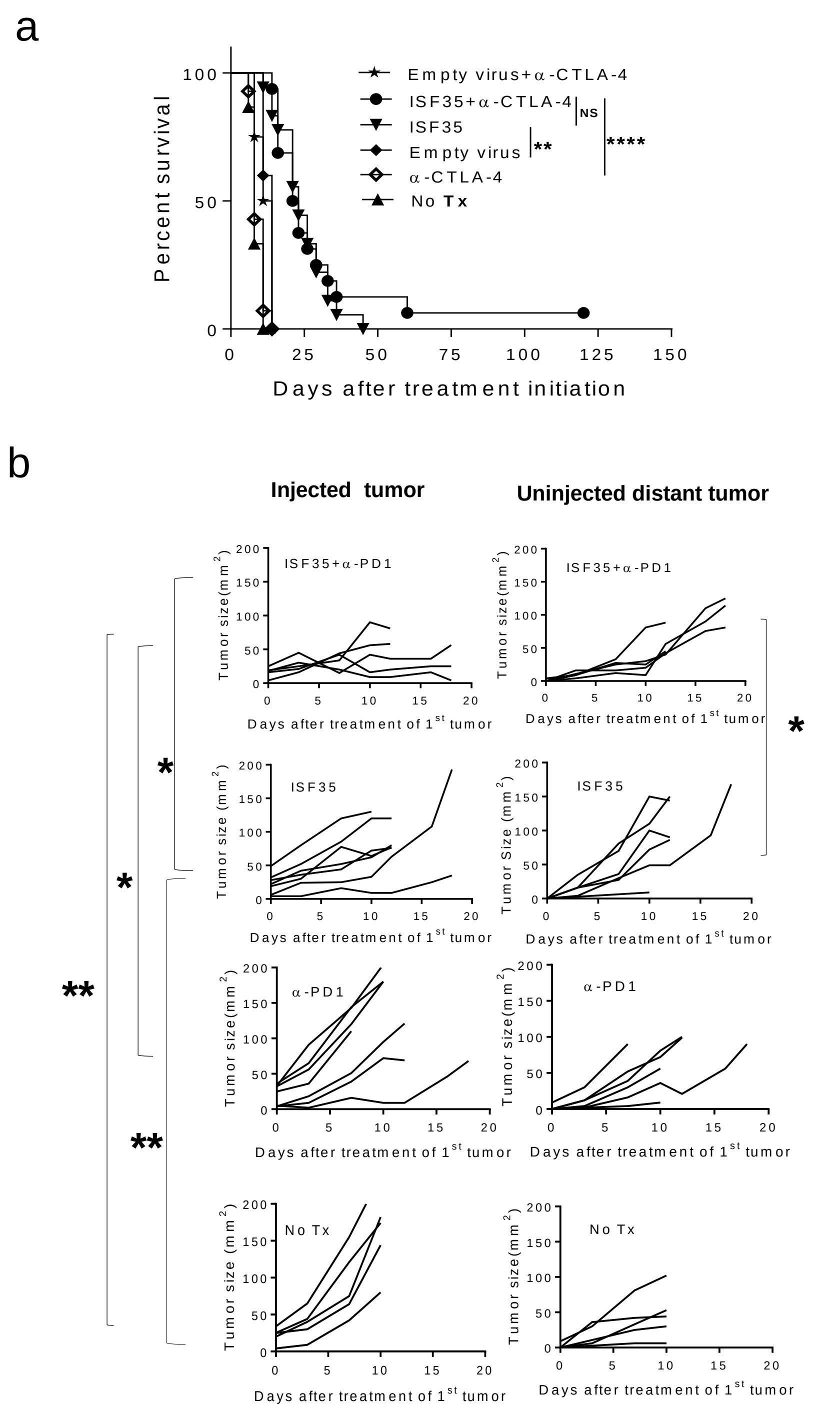

Supplementary Figure.2. Anti-tumor effect of intratumoral ISF35 and anti-PD-1 or anti-CTLA-4 antibody combination therapy. (a) Mice bearing 7-day s.c. B16.F10 tumors were treated as indicated, graph depicts long term survival(n=20) (b) Mice bearing 7 day old right flank tumor and 5 days old left flank tumor were treated only in the right flank tumor with ISF35 and received anti-PD-1 i.p. Graphs depict growth of ISF35-injected or uninjected opposite tumors. Data is analyzed by unpaired two-tailed t test. *p<0.05, **p<0.005, ***p<0.0005, ****p<0.0001 (n=5 to 7 mice). Error bars are SEM. Survival analysis was performed with the log-rank test
